# Supplementary material for: The Effect of a Personalized Approach to Patient Education on Heart Failure Self-Management
Source: J Pers Med. 2018 Nov 27;8(4):39. doi: 10.3390/jpm8040039 (PMC6313701; doi:10.3390/jpm8040039)
Supplement: Supplementary file 1 [file jpm-08-00039-s001.pdf]

Patient Education Tool

Heart failure is a condition which can cause extra fluid to build up in your lungs, feet, legs, or ankles.

This can make you feel short of breath, weak, or tired.

BIG VEIN (IVC) with extra SALT and FLUID in your body*

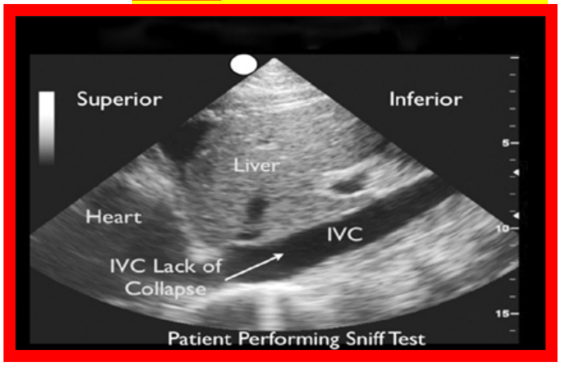

Figure 1

BIG VEIN (IVC) with NO extra SALT and FLUID in your body*

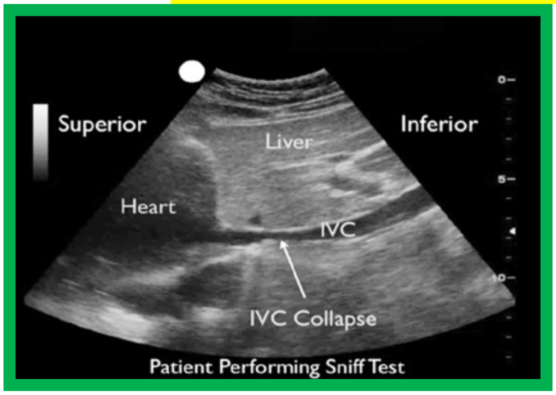

Figure 2

*Images courtesy of Journal of Emergency, Trauma and Shock www.jets.org)

HERE'S HOW YOUR BIG VEIN (IVC) LOOKS

Patient's personal IVC image to be printed and placed in this space.

Remember, we will call you on or about in about 2 weeks (_____) and in about 4 weeks (_______).

PATIENT EDUCATION TOOL - Heart Failure

With heart failure, sometimes too much fluid is kept in your lungs, feet, legs, or ankles.

Ultrasound picture of your big vein (IVC)

- A simple and easy test that does not hurt
- Picture of the big vein (IVC) carrying blood to the heart
- Shows if you have too much salt and fluid in your body

- If it is **large and plump,** you have **too much salt and fluid.**

- If it is **small and skinny,** this is **good**, and you don't have too much salt and fluid

Here are some things you can do to keep the extra fluid away.

- Don't salt your food.
- Don't eat foods that are high in salt.
- Stay away from potato chips, pretzels, popcorn, pickles, bacon, ham, hot dogs, and soy sauce.
- Stay away from canned foods, especially canned soups, and from canned and frozen main courses.
- Stay away from fast foods like McDonalds, Burger King, and Popeyes.
- Fresh fruits and vegetables are best.
- Read food labels. The total amount of sodium in your diet should be less than 2,000 mg per day.
- Take your medicines as directed by your doctor.
- Weigh yourself each day on a scale at the same time each day with the same type of clothes (or without clothes). If you gain weight quickly, especially more than 3 lbs in a day or 5 lbs in a week, call your doctor.

We hope the pictures of your big vein (IVC) may help you remember what you can do to keep the extra salt and fluid away.

JOHNS HOPKINS
MEDICINE
JOHNS HOPKINS
BAYVIEW MEDICAL CENTER
